# Transmission of ER stress response by ATF6 promotes endochondral bone growth

**DOI:** 10.1186/s13018-015-0284-7

**Published:** 2015-09-15

**Authors:** Zhangyuan Xiong, Rong Jiang, Peng Zhang, Xiaofeng Han, Feng-Jin Guo

**Affiliations:** Department of Cell Biology and Genetics, Core Facility of Development Biology, Chongqing Medical University, Chongqing, 400016 China; Laboratory of Stem Cells and Tissue Engineering, Chongqing Medical University, Chongqing, China

**Keywords:** ATF6, BMP2, UPR, Chondrocyte differentiation, Runx2, Bone growth

## Abstract

**Background:**

We reported earlier that X-box binding protein1 spliced (XBP1S), a key regulator of the unfolded protein response (UPR), as a bone morphogenetic protein 2 (BMP2)-inducible transcription factor, positively regulates endochondral bone formation by activating granulin-epithelin precursor (GEP) chondrogenic growth factor. Under the stress of misfolded or unfolded proteins in the endoplasmic reticulum (ER), the cells can be protected by the mammalian UPR. However, the influence of activating transcription factor 6 (ATF6), another transcriptional arm of UPR, in BMP2-induced chondrocyte differentiation has not yet been elucidated. In the current study, we investigate and explore the role of ATF6 in endochondral bone formation, focus on associated molecules of hypertrophic chondrocyte differentiation, as well as the molecular events underlying this process.

**Methods:**

High-cell-density micromass cultures were used to induce ATDC5 and C3H10T1/2 cell differentiation into chondrocytes. Quantitative real-time PCR, immunoblotting analysis, and immunohistochemistry were performed to examine (1) the expression of ATF6, ATF6α, collagen II, collagen X, and matrix metalloproteinase-13 (MMP13) and (2) whether ATF6 stimulates chondrogenesis and whether ATF6 enhances runt-related transcription factor 2 (Runx2)-mediated chondrocyte hypertrophy. Culture of fetal mouse bone explants was to detect whether ATF6 stimulates chondrocyte hypertrophy, mineralization, and endochondral bone growth. Coimmunoprecipitation was employed to determine whether ATF6 associates with Runx2 in chondrocyte differentiation.

**Results:**

ATF6 is differentially expressed in the course of BMP2-triggered chondrocyte differentiation. Overexpression of ATF6 accelerates chondrocyte differentiation, and the ex vivo studies reveal that ATF6 is a potent stimulator of chondrocyte hypertrophy, mineralization, and endochondral bone growth. Knockdown of ATF6 via a siRNA approach inhibits chondrogenesis. Furthermore, ATF6 associates with Runx2 and enhances Runx2-induced chondrocyte hypertrophy. And, the stimulation effect of ATF6 is reduced during inhibition of Runx2 via a siRNA approach, suggesting that the promoting effect is required for Runx2.

**Conclusions:**

Our observations demonstrate that ATF6 positively regulates chondrocyte hypertrophy and endochondral bone formation through activating Runx2-mediated hypertrophic chondrocyte differentiation.

## Background

Elaborate chondrogenesis is a convoluted biological process initiated by the growth of chondrocytes in the primary center of ossification. Besides the interactions between cells and growth factors, matrix proteins, and other environmental factors that mediate cellular signaling pathways, transcription of specific genes in a temporal-spatial manner also regulates the chondrocyte differentiation [1–3]. Bone morphogenetic protein 2 (BMP2) is one of the most important cytokines and plays several important roles in a variety of cellular functions ranging from embryogenesis, cell growth, and differentiation to bone development and the repair of bone fractures [4, 5]. It is known that BMP2 can activate unfolded protein response (UPR)-signaling molecules, such as BiP (binding immunoglobulin protein), CHOP (C/EBP homologous protein), ATF4 (activating transcription factor 4), and IRE1α (inositol-requiring enzyme-1α). Recently, another BMP2-signaling pathway in osteoblasts, mediated by the unfolded protein response of endoplasmic reticulum (ER) stress, was reported by Murakami et al [6].

ER stress activates a set of signaling pathways collectively termed as the unfolded protein response, which can mitigate the accumulation of misfolded or unfolded proteins in the endoplasmic reticulum [7–9]. The UPR is divided into three arms, including the PKR-like ER-resistant kinase (PERK), activating transcription factor 6 (ATF6), and IRE1α; the three together act to restrict new protein synthesis and increase the production of chaperones. ATF6 is an endoplasmic reticulum membrane-bound transcription factor that regulates various cellular functions [10–14].

We previously reported that BMP2 induces mild ER stress during chondrocyte differentiation and activates the IRE1α–XBP1 pathway. And then, X-box binding protein1 spliced (XBP1S) positively regulates endochondral bone formation by activating granulin-epithelin precursor (GEP) chondrogenic growth factor [13]. Jang et al. reported that BMP2 can activate UPR transducers, such as PERK, OASIS (old astrocyte specifically induced substance), and ATF6. BMP2 induces osteoblast differentiation through runt-related transcription factor 2 (Runx2)-dependent ATF6 expression, which directly regulates osteocalcin transcription. We also found that ATF6 was a positive regulator of ER stress-mediated apoptosis in chondrocyte differentiation [15]. However, whether ATF6 can influence the chondrocyte differentiation and endochondral bone growth has not yet been elucidated; specifically, the molecular mechanism underlying these processes remains unexplored. The purpose of this study was to investigate the role of ATF6 during the course of chondrogenesis and bone formation. We particularly focus on associated molecules of hypertrophic chondrocyte differentiation. In this study, we attempted to determine whether ATF6 is essential for skeletal development by using both in vitro and in vivo approaches. Our results support a novel role of ATF6 and endochondral bone growth through Runx2 transcription factor.

## Materials and methods

### Ethics statement

With the approval of the Chongqing Medical University Institutional Animal Care and Use Committee (permit number: SYXK 2007-0001, SCXK 2007-0002), all mice were housed under controlled temperatures in a 12-h light/dark cycle with easy access to food and water. This study was carried out in strict accordance with the recommendations in the Guide for the Care and Use of Laboratory Animals of the National Science Foundation of China. The protocol was approved by the Committee on the Ethics of Animal Experiments of Chongqing Medical University. All surgeries were performed under sodium pentobarbital anesthesia, and all efforts were made to minimize suffering.

### Plasmids and adenoviruses

The AdEasy adenoviral vector system (Invitrogen, Carlsbad, CA) was used to construct an adenovirus expressing ATF6 (Ad-ATF6). Briefly, ATF6 complementary DNA (cDNA) was inserted into the Xho I and Hind III sites in pAdTrack-cytomegalovirus (CMV) vector. The predigested recombinant adenovirus DNA was transfected into human embryonic kidney 293 cells. After collecting the medium supernatant that contains recombinant adenovirus, multiplicity of infection (MOI) for the recombinant adenovirus was determined according to the standard protocol. The expression of recombinant virus in infected ATF6 cells was tested by Western blotting with specific antibodies.

To generate ATF6 small interfering RNA (siRNA) expression constructs, siRNA corresponding to the coding sequence of the ATF6 gene (5′-AGCACCCAAGACTCAAACA ATTTT-3′) [15] was cloned into a pSES-HUS vector (an adenoviral shuttle vector expressing siRNA) according to the manufacturer’s instructions. Briefly, equimolar amounts of complementary sense and antisense strands were separately mixed, annealed, and slowly cooled to 10 °C in a 50-μl reaction buffer (100 mM NaCl and 50 mM HEPES, pH 7.4). The annealed oligonucleotides were inserted into the SfiI sites of pSES-HUS vector. All constructs were verified by nucleic acid sequencing; subsequent analysis was performed using BLAST software (National Institutes of Health).

Adenovirus Runx2 (Ad-Runx2) and siRunx2 were constructed, respectively, using methods described previously [13, 16].

### Cell culture

The micromass culture was performed as described previously [13, 17]. Briefly, multipotent murine CH10T1/2 cells were trypsinized and resuspended in Dulbecco’s modified Eagle’s medium (DMEM) with 10 % fetal bovine serum (FBS) at a concentration of 10^6^ cells/ml, and six drops of 100 μl of cells were placed in a 60-mm tissue culture dish (BD Biosciences), respectively. After 2-h incubation at 37 °C, 1 ml of DMEM containing 10 % FBS and BMP2 protein (300 ng/ml) was added. The medium was replaced approximately every 2–3 days. To test the effect of overexpression of ATF6 protein on chondrogenesis, C3H10T1/2 cells were infected with BMP2, BMP2 + Ad-ATF6 expression adenovirus, and control green fluorescent protein (GFP) adenovirus before micromass culture.

Mouse chondrogenic ATDC5 cells were maintained in a medium consisting of a 1:1 mixture of DMEM and Ham’s F-12 medium (Flow Laboratories, Irvine, UK) containing 5 % FBS (Invitrogen), 10 mg/ml human transferrin (Roche Applied Science), and 30 nM sodium selenite (Sigma) at 37 °C in a humidified atmosphere of 5 % CO_2_ in air. The ATDC5 cells were seeded at a density of 3 × 10^5^ cells/well in 6-well cell culture plates (Corning). The medium was replaced every other day. For adenovirus (Ad-ATF6 or Ad-GFP) infection and Ad-ATF6 siRNA, Ad-RFP infection, the same protocol as used with C3H10T1/2 cells was followed.

### Immunohistochemistry

Sections of postcoital day 15.5 and 17.5 embryos, newborn mice, and day 10 mice were deparaffinized, rehydrated, and placed in Tris buffer (10 mM Tris-HCl (pH 8.0), 150 mM NaCl). Serum block was applied for 30 min at room temperature before incubation of the primary antibody. Anti-mouse ATF6 (Bio-Legend) was diluted 1:50, and sections were incubated at room temperature for 2 h. For detection, biotinylated secondary antibody and horseradish peroxidase (HRP)-streptavidin complex (Santa Cruz Biotechnology, Inc., Santa Cruz, CA) were used. HRP substrate was used for visualization, and sections were then counterstained with Mayer’s hematoxylin.

### Quantitative real-time PCR

To examine the effects of ATF6 on the chondrocyte differentiation, C3H10T1/2 or ATDC5 cells were plated at a density of 3 × 10^5^ cells/well in 6-well tissue culture plates. One microgram of Ad-ATF6 or siATF6 was then infected into these cells, respectively.

After 48 h, total RNAs were isolated using the RNeasy mini kit (Qiagen, Hilden, Germany) and reverse transcribed into cDNA. Real-time PCR was performed with an ABI 7400 system using the TaqMan EZ RT-PCR kit according to the manufacturer’s protocol. TaqMan primers and probes were derived from the commercially available TaqMan assay-on-demand gene expression products. We select GAPDH as the endogenous control for the real-time PCR relative quantification analysis. PCR cycling conditions were as follows: initial incubation step of 2 min at 50 °C, reverse transcription of 60 min at 60 °C and 94 °C for 2 min, followed by 40 cycles of 15 s at 95 °C for denaturation and 2 min at 62 °C for annealing and extension.

In the case of collagen II, collagen X, and matrix metalloproteinase-13 (MMP13), real-time PCR was run using the SYBR Green PCR kit, and the following primers were used: sense (3′-AACGAGAACGACGAG GTGGT-5′) and antisense (3′-AAAGGAGGCAGATGACAG GTGAC-5′) for collagen II, sense (3′-TACCACGTGCATGTGAAAGG-5′) and antisense (3′-GGAGCCACTAGGAATC CTGAG-5′) for collagen X, and sense (3′-CAGTTTGCAGAGCGCTACCTG AG-5′) and antisense(3′-GGGTTCCTGATGTGGGTG-5′) for MMP13. The following pair of oligonucleotides was used as internal controls: 5′-ACCACAGTCCATGCCATCAC-3′ and 5′-TCCACCACCCTGTTGCTG TA-3′ for GAPDH.

### Immunoblotting analysis

To examine the expression of ATF6 and collagen X protein in the course of chondrogenesis, total cell extracts prepared from micromass cultures of C3H10T1/2 cells in the presence of 300 ng/ml recombinant BMP2 protein were mixed with 5 × sample buffer (312.5 mM Tris-HCl (pH 6.8), 5 % β-mercaptoethanol, 10 % SDS, 0.5 % bromphenol blue, 50 % glycerol). Proteins (1 μg/μl) were resolved on a 10 % SDS-polyacrylamide gel and electroblotted onto a nitrocellulose membrane. After blocking in 10 % nonfat dry milk in Tris buffer, saline Tween 20 (10 mM Tris-HCl (pH 8.0), 150 mM NaCl, 0.5 % Tween 20), blots were incubated with either mouse monoclonal anti-ATF6 antibody (diluted 1:500; Bio-Legend) or rabbit polyclonal anti-collagen X (diluted 1:500; Santa Cruz Biotechnology, Inc.) for 1 h. After washing, the respective secondary antibody (HRP-conjugated anti-mouse immunoglobulin or HRP-conjugated anti-rabbit immunoglobulin (Sigma), both 1:1000 dilution) was added, and bound antibody was visualized using an enhanced chemiluminescence system (Amersham Biosciences).

### Culture of fetal mouse bone explants

Fetal mouse metatarsals were dissected from 15-day-old mouse embryos and cultured in DMEM (Gibco, Carlsbad, CA, USA) containing 1 % heat-inactivated fetal calf serum (Invitrogen) and 100 U penicillin-streptomycin per milliliter in the absence or presence of various stimuli for 5 days, as indicated in Fig 2.

For alizarin red and Alcian Blue staining (alizarin red staining for the detection of mineralized bone and Alcian Blue staining for the detection of cartilage), the explants were placed in 4 % paraformaldehyde in phosphate-buffered saline for overnight fixation. Subsequently, explants were placed in staining solution (0.05 % alizarin red, 0.015 % alcian blue, 5 % acetic acid in 70 % ethanol) for 45–60 min. Digital images of stained bones were analyzed. For safranin O–fast green staining (safranin O staining for the detection of cartilage and fast green staining for subchondral bone and extracellular matrix), explants were fixed in 96 % alcohol and processed for paraffin embedding. Sections were stained with 0.1 % safranin O (orange stain) to evaluate cartilage matrices and with 0.03 % fast green to evaluate morphological features as previously described [13, 18].

### Coimmunoprecipitation

Approximately 500 mg of cell extract proteins were prepared from C3H10T1/2 cells treated with BMP2 for 5 days. Then, micromass culture of C3H10T1/2 cells were incubated with anti-Runx2 (20 mg/ml; Santa Cruz Biotechnology, Inc.) or control rabbit IgG (25 mg/ml) antibodies for 1 h, followed by incubation with 30 ml of protein A-agarose (PerkinElmer Life Sciences) at 4 °C overnight. After washing five times with immunoprecipitation buffer, bound proteins were released by boiling in 20 ml of 2 × SDS loading buffer for 3 min. Released proteins were examined by Western blotting with anti-ATF6 antibody, and the signal was detected using the ECL chemiluminescent system.

### Statistical test

The statistical analysis was performed with SPSS 10.0.1 software for Windows. Data were expressed as mean ± SD from at least three independent experiments. Data for multiple variable comparisons were analyzed by one-way analysis of variance. *p* values of <0.05 were deemed statistically significant.

## Results

### Differential expression of ATF6 in the course of chondrogenesis

**Fig. 1:**
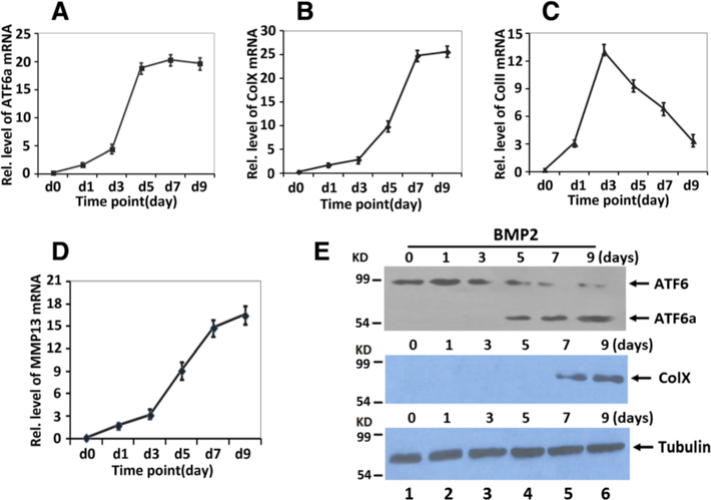
Expression of ATF6 and ATF6a in the course of chondrogenesis in a micromass culture of ATDC5 cells. **a**–**d** Real-time PCR assay. Total RNA was prepared from micromass cultures of ATDC5 cells in the presence of 300 ng/ml recombinant BMP2 for various time points, as indicated, and the mRNA expression of ATF6a, collagen II, collagen X, MMP13, and GAPDH (serving as an internal control) were examined by real-time PCR. **e** Western blotting assay. After incubation of micromass cultures of ATDC5 cells with 300 ng/ml BMP2 for the times indicated, the cells were lysed, and 40-mg protein samples were assayed for ATF6, ATF6a, collagen X, and tubulin (serving as an internal control) by Western blotting with anti-ATF6, anti-collagen X, or anti-tubulin, respectively. The *arrow* is protein ATF6, ATF6a, and collagen X, respectively

We next studied ATF6 and ATF6a expression profiles during chondrocyte differentiation using the ATDC5 cell line, a pluripotent murine stem cell line that is a well-established in vitro cell model. Cells were harvested at various time points followed by real-time PCR for measurements of ATF6a, collagen II, collagen X, and MMP13 (Fig. 1a–d). As revealed in Fig. 1a–d, the mRNA level of ATF6a was relatively low until day 5, when it had doubled, and thereafter remained at high levels during the differential stage, although collagen II declined after 3 days of BMP2 treatment. Note that indication of the high level of ATF6a was 2 days earlier than that of collagen X and MMP13, two specific markers for hypertrophic chondrocytes, therefore suggesting that ATF6a may regulate chondrocyte hypertrophy through collagen X and MMP13 expression.

Then, we examined the protein expression profiles of ATF6 and ATF6a during chondrocyte differentiation. BMP2 induces mild ER stress, and then ATF6, as a 90-kDa protein (p90ATF6) in previous non-ER stress environment, is directly converted to a 50-kDa protein (p50ATF6, ATF6a) in ER-stressed cells. As revealed in Fig. 1e, ATF6 undergoes proteolysis and splicing after BMP2 stimulation. ATF6a protein was not detected until day 5 in BMP2-induced chondrocyte differentiation of ATDC5 cells. The expression of collagen X was also immune positive at day 7, indicating that ATF6a expression is prehypertrophic and hypertrophic chondrocyte-specific. The ER stress-induced ATF6 proteolysis occurs in BMP2 stimulation day 5. More significantly, ATF6a expression was 2 days earlier than that of collagen X.

### ATF6 expression patterns in the chondrocytes of the growth plate in vivo

**Fig. 2:**
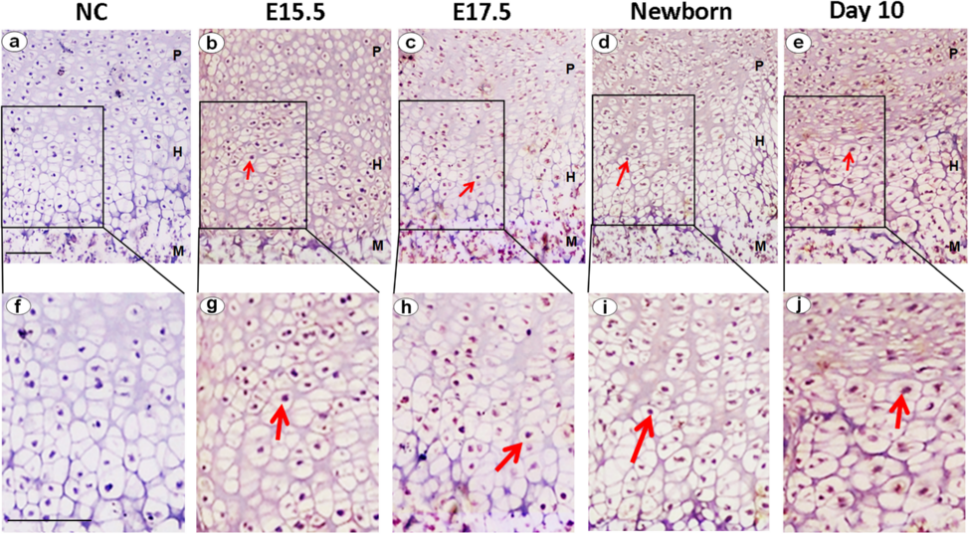
Expression of ATF6 in the growth plate chondrocytes in vivo. ATF6 immunohistochemistry in tibial growth plates of postcoital day 15.5 mouse embryo (E15.5; **b**, **g**), postcoital day 17.5 mouse embryo (E17.5; **c**, **h**), newborn (**d**, **i**) and day 10 old (**e**, **j**). Microphotographs are shown of sections stained with anti-ATF6 antibody (*brown*) and counterstained with hematoxylin (*blue*). Immunostaining reveals positive nuclear staining in the entire chondrogenic developmental stages in both proliferating and hypertrophic zones, and the *scale bars* represent 100 μm. The *red arrow* is protein ATF6. *NC* (**a**, **f**) negative control, *P* proliferating chondrocytes, *H* hypertrophic chondrocytes, *M* bone metaphysis

It is reported that ER stress signal molecules were associated with chondrogenesis [19–21]. In this study, we sought to determine whether ATF6, an important transcription factor in ER stress, was also involved in the chondrocyte differentiation. First, we examined the temporal and spatial expression pattern of ATF6 during skeletal development using an immunostaining assay at multiple time points, including embryonic day(E) E15.5, and E17.5, as well as postnatal developmental stages (newborn and 10 days). As revealed in Fig. 2, ATF6 demonstrated prominent expression throughout the growth plate chondrocytes at postcoital days 15.5 (b, g) and 17.5 (c, h), and in the newborn (d, i) and 10-day mice (e, j), NC (a, f) is negative control. These results suggested that the expression profile of ATF6 is closely linked to the entire chondrogenic period, including proliferating zone (P) and hypertrophic zone (H), especially highly expressed in the hypertrophic chondrocyte stage.

### Overexpressing ATF6 activates chondrogenesis in vitro and endochondral bone formation ex vivo

**Fig. 3:**
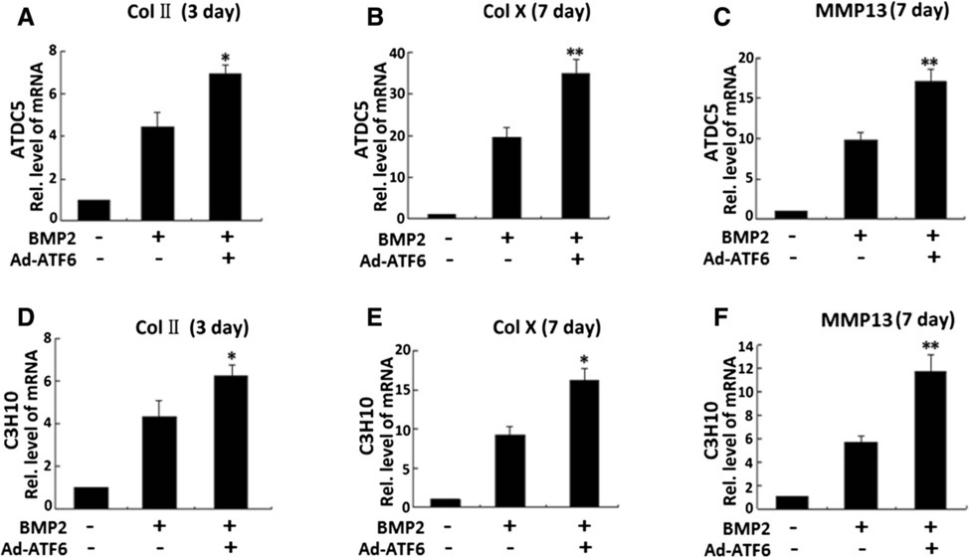
ATF6 stimulates chondrogenesis in vitro. Comparisons of ATF6 and BMP2 in stimulations of chondrogenesis of ATDC5 cells (**a**–**c**) and C3H10T1/2 cells (**d**–**f**). Micromass cultures of ATDC5 and C3H10T1/2 cells were incubated in the absence (CTR, Ad-GFP, serves as a control) or presence of either 300 ng/ml BMP2, Ad-ATF6 (MOI = 50), or Ad-ATF6 (MOI = 50) + BMP2 for 3 or 7 days, and the mRNA levels of Col II (**a**, **d**), Col X (**b**, **e**), and MMP13 (**c**, **f**) were determined using real-time PCR. The units are arbitrary, and the normalized values were calibrated against control (*CTR*), here given the value of 1. The *asterisk* indicates significant increase or decrease from control (*p < 0.05, **p < 0.01)

High-cell-density micromass cultures can differentiate specifically to the cartilage lineage at high yields when exposed to chondroinductive factors such as a well-documented growth factor BMP2 [1–3]. We next sought to determine the role of ATF6 and BMP2 (300 ng/ml) during chondrogenesis in micromass cultures of ATDC5 cells and C3H10T1/2 cells [22, 23]. Briefly, the high-density culture system was incubated in the absence (control (CTR)) or presence of Ad-ATF6 or 300 ng/ml BMP2 (serving as a positive control) for induction of chondrocyte differentiation. Cells were harvested at various time points, 3 or 7 days. Therefore, both ATDC5 and C3H10T1/2 cells have the potential to become chondrocytes, making them a valuable in vitro correlate for studying the mechanisms of chondrogenesis. Chondrogenesis was monitored by analyzing the expressions of marker genes specific for chondrocytes. ATDC5 or C3H10T1/2 cells were treated with BMP2, Ad-ATF6 + BMP2, and control GFP (Ad-GFP), respectively, and then RNA was extracted every other day for real-time PCR. As revealed in Fig. 3, chondrocyte differentiation was monitored by examining the expression of collagen II, collagen X, and MMP13, three marker genes widely used for chondrocyte maturation and hypertrophy [2, 24, 25]. As for BMP2, ATF6 markedly induced the expression of collagen II, collagen X, and MMP13. Besides, in Ad-ATF6 + BMP2-treated ATDC5 cells, the expressions of collagen II, collagen X, and MMP13 were increased to 1.7-fold, 1.9-fold, and 1.9-fold, respectively, compared with those in BMP2-treated cells (Fig. 3a–c). And, in Ad-ATF6 + BMP2-treated C3H10T1/2 cells, the expressions of collagen II, collagen X, and MMP13 were increased to 1.6-fold, 2.0-fold, and 2.1-fold, respectively, compared with those in BMP2-treated cells (Fig. 3d–f). The clearly enhanced expressions of three marker genes (collagen II, collagen X, and MMP13) were observed in Ad-ATF6 + BMP2-treated cells compared with those in BMP2-treated cells, suggesting that overexpression of ATF6 can enhance BMP2-induced chondrocyte differentiation.

Thus, ATF6 is a positive mediator for chondrocyte differentiation and hypertrophy. The difference was statistically significant between the Ad-ATF6 + BMP2 group and BMP2 group (*p < 0.05, **p < 0.01).

**Fig. 4:**
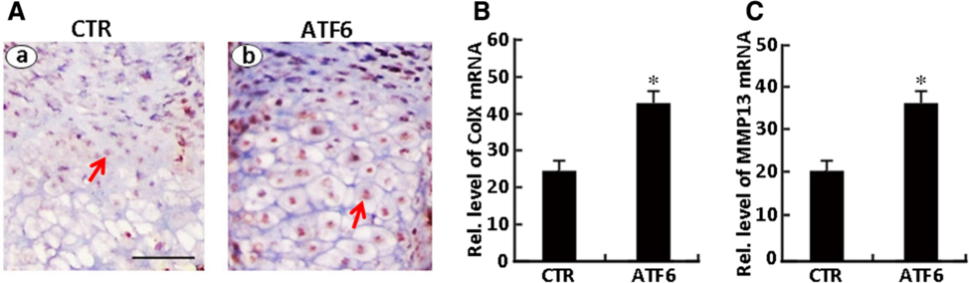
Expression of ATF6 in the growth plate chondrocytes in vivo. **a** Immunohistochemistry of ATF6 in the section of long bone from a 15-day-old mouse embryo. Metatarsals from 15-day-old mouse embryos were cultured in the absence (Ad-GFP, serves as a control) or presence of Ad-ATF6 (MOI = 50) for 7 days, then processed for paraffin embedding and sections were performed. The section was stained with anti-ATF6 polyclonal antibody (*brown*) and counterstained with Mayer’s hematoxylin (*blue*). Immunostaining for ATF6 demonstrates localization of strongly immunopositive chondrocytes in the lower proliferative/upper hypertrophic zones of the growth plate. The expression of ATF6 dramatically increased after infected with Ad-ATF6, the *scale bars* represent 100 μm. The *red arrow* is protein ATF6. **b**, **c** Metatarsals from 15-day-old mouse embryos were cultured in the absence (Ad-GFP) or presence of Ad-ATF6 (MOI = 50) for 5 days, and the mRNA levels of Col X (**b**) and MMP13 (**c**) were determined using real-time PCR. The normalized values were calibrated against control (*CTR*). The *asterisk* indicates significant increase or decrease from control (*p < 0.05)

**Fig. 5:**
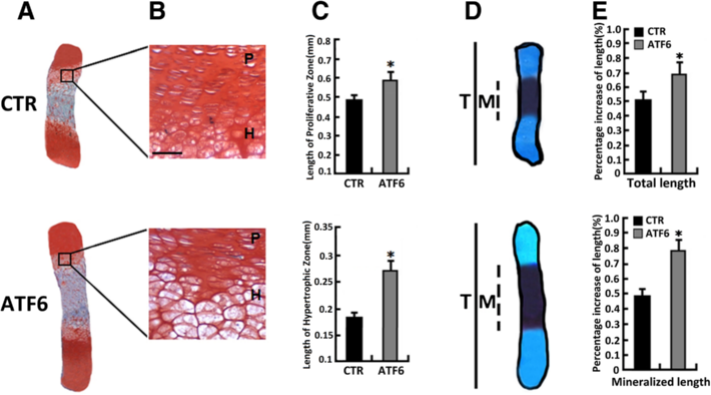
ATF6 stimulates chondrocyte hypertrophy, mineralization, and endochondral bone growth. **a**, **b** Safranin O/fast green staining of metatarsal bones. Metatarsals from 15-day-old mouse embryos were cultured in the absence (CTR) or presence of Ad-ATF6 (MOI = 50) for 7 days and stained with safranin O/fast green, shown in low-power (**a**) and high-power (**b**) microphotographs. **c** Quantification of the length of the proliferating (*P*) zone and hypertrophic (*H*) zone in growth plates of metatarsal bones. *p < 0.05 versus control by paired Student’s *t* test. **d** Alizarin red/Alcian Blue staining of metatarsals. Explants were fixed and processed for staining. A representative photograph of an explanted metatarsal is presented. **e** Percentage changes in total (*T*) and mineralization (*M*) length of metatarsal bones. Percentage changes in bone length were calculated as (length at d5-length at d0)/length at d0. *p < 0.05 versus control; scale bar = 100 μm. Error bars, SD

The effect of ATF6 on endochondral bone formation was then studied in an ex vivo model of 15-day-old fetal mouse metatarsal bones. In a 7-day culture period of Ad-ATF6 (MOI = 50), these explants underwent all sequential stages of endochondral bone formation. Firstly, ATF6 expression was detected and enhanced in metatarsals from 15-day-old mouse embryos infected with Ad-ATF6 (Fig. 4a) compared with CTR; then, the mRNA levels of Col X (b), MMP13 (c) were determined using real-time PCR. As shown in Fig. 4b, c, the mRNA levels of Col X and MMP13 were increased after the 15-day-old mouse embryos were infected with Ad-ATF6, suggesting that ATF6 obviously stimulated chondrocyte hypertrophy and differentiation. Besides, as revealed in Fig. 5, metatarsals from 15-day-old mouse embryos were cultured in the absence (CTR) or presence of Ad-ATF6 (MOI = 50) for 7 days; then, stained with safranin O/fast green and alizarin red/Alcian Blue staining of metatarsals, the result showed that the length of proliferative zone and hypertrophic zone of the Ad-ATF6 infection group was elongated compared with CTR, and the percentage increase of total length and mineralized length of the Ad-ATF6 infection group was also improved compared with CTR. It was demonstrated that ATF6 significantly stimulated chondrocyte hypertrophy, mineralization, and bone length. The difference was statistically significant between the Ad-ATF6 group and BMP2 group (*p < 0.05).

### Knockdown of ATF6 inhibits hypertrophic chondrocyte differentiation

**Fig. 6:**
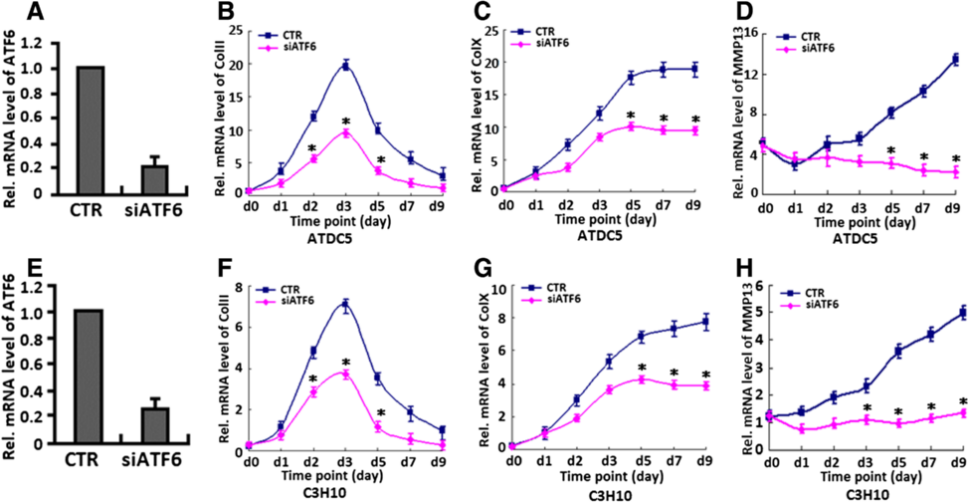
Knockdown of ATF6 using the siRNA approach inhibits chondrocyte hypertrophy, as revealed by collagen II, collagen X, and MMP13 expression. **a**, **e** siRNA against ATF6 mRNA efficiently inhibited expression of endogenous ATF6 in both ATDC5 (**a**) and C3H10 cells (**e**). Cells were infected with either Ad-ATF6 siRNA or control adenovirus (*CTR*), and total RNA was collected for real-time PCR. Expression of ATF6 was normalized against the GAPDH endogenous control. The normalized values were then calibrated against the control value, here set as 1. *p < 0.05. **b**, **f** Repression of ATF6 clearly abolished BMP2-induced Col II expression in ATDC5 (**b**) and C3H10 (**f**) cells. Transcript levels of Col II were detected by real-time PCR analysis of RNA isolated from micromass cultures of ATDC5 (**b**) or C3H10 (**f**) cells infected with siATF6 (MOI = 50) or control adenovirus in the presence of 300 ng/ml BMP2 at various time points, as indicated. *p < 0.05. **c**, **g** Repression of ATF6 obviously inhibits BMP2-induced Col X expression in ATDC5 (**c**) and C3H10 (**g**) cells. Transcript levels of Col X were detected by real-time PCR analysis of RNA isolated from micromass cultures of ATDC5 (**c**) or C3H10 (**g**) cells infected with siATF6 (MOI = 50) or control adenovirus in the presence of 300 ng/ml of BMP2 at various time points, as indicated. *p < 0.05. **d**, **h** Repression of ATF6 largely abolished BMP2-induced MMP13 expression in ATDC5 (**d**) and C3H10 (**h**) cells. Transcript levels of MMP13 were detected by real-time PCR analysis of RNA isolated from micromass cultures of ATDC5 (**d**) or C3H10 (**h**) cells infected with siATF6 (MOI = 50) or control adenovirus in the presence of 300 ng/ml BMP2 at various time points, as indicated. *p < 0.05. Error bars, SD

Having known that ATF6 can enhance hypertrophic chondrocyte differentiation, we next determined whether endogenous ATF6 is required for chondrocyte hypertrophy by knocking down ATF6 using the siRNA approach in ATDC5 cells and C3H10 cells. A real-time PCR was performed to verify the RNA level of ATF6 in both cells. As shown in Fig. 6a, e, infection with siATF6 adenovirus resulted in ~79 and 74 % reduction in ATF6 mRNA in ATDC5 cells and C3H10 cells, respectively. Micromass cultures of ATDC5 cells or C3H10 cells infected with siATF6 adenovirus or control adenovirus (CTR) were treated with BMP2 for various time points. As shown in Fig. 6, knockdown of ATF6 obviously abolished the collagen II (Fig. 6b, f), collagen X (Fig. 6c, g), and MMP13 (Fig. 6d, h) induction during chondrocyte differentiation. These findings clearly indicated that knockdown of ATF6 via siRNA clearly inhibited BMP2-induced chondrocyte differentiation and hypertrophy, and endogenous ATF6 is required for hypertrophic chondrocyte differentiation.

### ATF6 associates with Runx2 during the course of chondrocyte differentiation

**Fig. 7:**
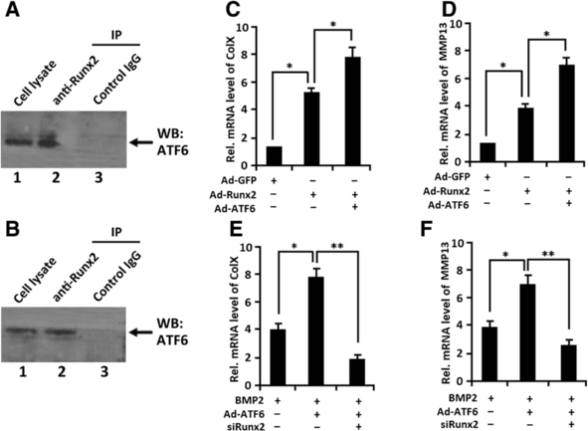
ATF6 associates with Runx2 in chondrogenesis and ATF6 enhances Runx2-mediated chondrocyte hypertrophy. **a** ATF6 binds to Runx2 inhypertrophic chondrocyte differentiation of C3H10T1/2 cells treated with BMP2 5 days. Cells lysates prepared from micromass culture of C3H10T1/2 cells treated with BMP2 5 days were collected and were incubated with either control IgG (*lane 3*) or Runx2 antibodies (*lane 2*), followed by protein A-agarose. The immunoprecipitated (*IP*) protein complex and cell extracts (*lane 1*; serving as a positive control) were examined by Western blotting with anti-ATF6 antibody. The *arrow* is protein ATF6. **b** ATF6 binds to Runx2 in hypertrophic chondrocyte differentiation of C3H10T1/2 cells treated with BMP2 7 days. Cell lysates prepared from micromass culture of C3H10T1/2 cells treated with BMP2 7 days were collected and analyzed, respectively, as described in **a**. The *arrow* is protein ATF6. **c** ATF6 enhanced RUNX2-dependent Col X expression in C3H10T1/2 cells. Transcript levels of Col X were detected by real-time PCR analysis of RNA isolated from C3H10T1/2 cells infected with either Ad-GFP (control), Ad-RUNX2 (MOI = 50), or Ad-ATF6 + Ad-RUNX2, as indicated. *p < 0.05. **d** ATF6 enhanced RUNX2-dependent MMP13 expression in C3H10T1/2 cells. Transcript levels of MMP13 were detected by real-time PCR analysis of RNA isolated from C3H10T1/2 cells infected with Ad-GFP(control), Ad-RUNX2 (MOI = 50), or Ad-ATF6 + Ad-RUNX2, as indicated. RNA was extracted as described in **a**, and MMP13 mRNA was detected by real-time PCR. *p < 0.05. **e** siRUNX2 decreases BMP2-induced Col X expression stimulated by ATF6 in C3H10T1/2 cells. Transcript levels of Col X were detected by real-time PCR analysis of RNA isolated from micromass culture of C3H10T1/2 cells infected with either BMP2, BMP2 + Ad-ATF6(MOI = 50), or BMP2 + Ad-ATF6+ siRUNX2(MOI = 50), as indicated. *p < 0.05,**p < 0.01. **f** siRUNX2 decreases BMP2-induced MMP13 expression stimulated by ATF6 in C3H10T1/2 cells. Transcript levels of MMP13 were detected by real-time PCR analysis of RNA isolated from micromass culture of C3H10T1/2 cells infected with either BMP2, BMP2 + Ad-ATF6, or BMP2 + Ad-ATF6 + siRUNX2, as indicated. *p < 0.05,**p < 0.01. Error bars, SD

We next sought to elucidate the molecular mechanism by which ATF6 mediates chondrocyte hypertrophy by determining whether ATF6 also associates with Runx2 in hypertrophic chondrocyte differentiation. To test whether ATF6 binds to Runx2 in micromass culture of C3H10T1/2 cells treated with BMP2 for 5 days, a coimmunoprecipitation assay was performed. Briefly, extracts from micromass culture C3H10T1/2 cells treated with BMP2 for 5 days were first incubated with control IgG (negative control) or anti-Runx2 antibodies, and the immunoprecipitated complexes were detected by Western blotting with anti-ATF6 antibody. An ATF6-specific band was present in the immunoprecipitated complexes brought down by anti-Runx2 (Fig. 7a, lane 2) but not by control antibodies, demonstrating that ATF6 specifically associates with Runx2 in C3H10T1/2 cells treated with BMP2 for 5 days.

Next to examine whether ATF6 binds to Runx2 in, micromass culture of C3H10T1/2 cells treated with BMP2 for 7 days, and a coimmunoprecipitation assay was performed again. First, extracts from C3H10T1/2 cells treated with BMP2 for 5 days were incubated with control IgG (Fig. 7b, lane 3) or anti-Runx2 antibodies (Fig. 7b, lane 2), and the immunoprecipitated complexes were detected by Western blotting with anti-ATF6 antibody. The result showed that ATF6 can bind to Runx2 in C3H10T1/2 cells treated with BMP2 for 7 days, indicating that ATF6 and Runx2 form a protein complex in BMP2-induced chondrogenesis. Together, these results indicate that ATF6 and Runx2 can form a protein complex in hypertrophic chondrocyte differentiation.

### ATF6 activates chondrogenesis and endochondral bone formation is required for Runx2

All is known that Runx2 is required for hypertrophic chondrocyte differentiation and activates collagen X expression, MMP13, and IHH expression [13, 26]. To determine whether ATF6 affects Runx2-dependent chondrocyte hypertrophy, micromass culture of C3H10T1/2 cells were infected with adenovirus encoding ATF6 (Ad-ATF6), Runx2 (Ad-Runx2), or a combination, and then RNA was extracted at day 7 for real-time PCR. As shown in Fig. 7c, d, markedly enhanced expressions of collagen X and MMP13 in Ad-ATF6 + Ad-Runx2-infected cells were observed compared with those in AdRunx2-infected cells. It was demonstrated that ATF6 effectively increased the collagen X and MMP13 expression induced by Runx2.

In addition, we also detected whether ATF6 stimulation effect is required for Runx2, micromass culture C3H10T1/2 cells were treated with BMP2, adenovirus encoding ATF6 (Ad-ATF6), Ad-Runx2 siRNA (si-Runx2), or a combination, and then RNA was extracted at day 7 for real-time PCR. As shown in Fig. 7e, f, remarkable enhanced expressions of collagen X and MMP13 in (BMP2 + Ad-ATF6)-treated cells were observed compared with those in BMP2-treated cells, suggesting that ATF6 obviously increased the collagen X and MMP13 expression induced by BMP2. However, the expression of collagen X and MMP13 was reduced in (BMP2 + Ad-ATF6 + siRunx2)-infected cells. The promoting effect of ATF6 is clearly inhibited after siRunx2 infection. It demonstrated that ATF6 activates chondrogenesis and endochondral bone formation and the promoting effect of ATF6 depends on Runx2.

## Discussion

The endoplasmic reticulum is a central cellular organelle responsible for the synthesis, folding, and posttranslational modifications of proteins destined for the secretory pathway. An overload of protein folding in the ER disrupts ER homeostasis, causing ER stress [27, 28]. Accumulated evidences indicate a physiological role of UPR during developmental processes. Furthermore, UPR leads to the ATF6 cleavage by the proteases in the Golgi complex. Then, the cleaved N-terminal ATF6 cytoplasmic domain is released from the Golgi membrane to the nucleus to regulate transcription. In mammalian cells, the UPR plays a fundamental role in maintaining cellular homeostasis and is therefore at the center of many normal physiological and pathological responses. Many studies have shown that factors influencing cell survival and differentiation are activated during ER stress [29–31], but how such changes impact differentiation programs in chondrocytes is poorly understood. Therefore, to test a link between the ATF6 branch of the UPR and cartilage development, we focused on the role of ATF6 in endochondral bone growth as well as the molecular mechanism.

ATF6 is a multifunctional protein involved in the control of cell proliferation and the differentiation of numerous types of cells and tissues including skeletal muscle myotubes, cardiac myocytes, hepatocyte, and osteoblasts [14, 32–36]. The current study sought to elucidate the role of ATF6 in regulating the chondrocyte differentiation, with the special focus on the molecular mechanism underlying this process. BMP2 induces mild ER stress, and then ATF6 undergoes proteolysis and produces ATF6a after BMP2 stimulation. The ATF6 and ATF6a expression profiles were different in the course of chondrogenesis in vitro. Both of them demonstrated prominent expression in the entire growth plate chondrocyte population in vivo. Real-time PCR result showed that not until day 5 did the mRNA level of ATF6 become relatively high, and at day 7, it tripled and thereafter remained at high levels during the late differential stage. The difference between mRNA and the protein of ATF6 during chondrogenesis suggests that posttranscriptional regulations, such as mRNA splice, translation and stability, and protein degradation, might also be important in the control of ATF6 expression during chondrocyte differentiation.

It is known that ATF6 is an ER membrane-bound bZIP transcription factor whose structure and mode of action is similar to those of OASIS. Under normal conditions, ATF6 interacts with the ER protein chaperone BiP/GRP78 and is retained in the ER membrane [6, 12, 37]. However, upon the accumulation of unfolded or misfolded proteins in the ER lumen, ATF6 is released from BiP and, as previously discussed, cleaved ATF6 will be transported to the nucleus [14, 33, 34]. In this study, we found that ATF6 functions as a positive regulator of chondrocyte differentiation, because overexpression of ATF6 enhances, whereas knockdown of ATF6 abolishes BMP2-induced chondrocyte differentiation and hypertrophy, as assayed by the expression of collagen II, collagen X, and MMP13, three marker genes for chondrocyte differentiation. The in vitro and in vivo studies support the concept that ATF6 is a potent stimulator of chondrocyte differentiation, mineralization, and endochondral bone growth.

Further, we sought to clarify the molecular mechanism of how to regulate chondrocyte differentiation by ATF6. As noted, Runx2 is a transcription factor that belongs to the Runx family. It is an essential central regulator of osteoblast differentiation and hypertrophic chondrocyte formation. Runx2 regulates the activity of their target genes by binding to the respective promoter or enhancer elements in a sequence-specific manner. A large number of regulatory proteins bind to the C-terminus of Runx2 to modify its transcriptional function. Besides, Runx2 can also bind to multiple recognition sites in the collagen X promoter and activate collagen X reporter constructs [38, 39]. Growing evidences demonstrate that a variety of Runx2-binding proteins regulates the activity of Runx2 and plays important roles in skeletogenesis [40–43]. Here, our data showed that ATF6 associates with Runx2 and enhances Runx2-induced hypertrophic chondrocyte differentiation. Firstly, ATF6 and Runx2 can form a protein complex in chondrocyte differentiation, and then overexpression of ATF6 can enhance the collagen X and MMP13 expression and then increases Runx2-dependent hypertrophic chondrocyte differentiation. In addition, the promoting effect of ATF6 is clearly reduced when Runx2 is inhibited via siRNA. It is suggested that the stimulation effect of ATF6 is required for Runx2. The results suggested that ATF6 is a cofactor of Runx2 in regulating hypertrophic chondrocyte differentiation. On the basis of our earlier findings [13, 15, 16, 18] and the results of this study, we conclude that ATF6 could positively regulate chondrocyte differentiation and endochondral bone formation, and this facilitating effect depends on Runx2.

## Summary

In a word, ATF6 positively regulates hypertrophic chondrocyte differentiation and endochondral bone formation through activating Runx2-mediated hypertrophic chondrocyte differentiation. Collectively, this study identifies that ATF6, as a new regulatory factor in the complex networks, regulates growth plate chondrocyte differentiation and endochondral bone formation. This study provides novel insights into the role of ATF6 in regulating chondrocyte differentiation and endochondral bone formation and sheds light on the molecular mechanism by which ATF6 positively regulates chondrogenesis, i.e., ATF6 increases chondrocyte differentiation and endochondral bone formation through activating Runx2 and endogenous Runx2 is required for ATF6-stimulated chondrocyte hypertrophy, mineralization, and endochondral bone growth. The elucidation of ATF6’s role and molecular events involved in chondrocyte differentiation will better our understanding of normal cartilage development and the pathogenesis of cartilage disease.

## Abbreviations

ATF6: activating transcription factor 6
BMP2: bone morphogenetic protein 2
IRE1α: inositol-requiring enzyme 1α
PERK: PKR-like ER-resistant kinase
UPR: unfolded protein response
XBP1S: X-box binding protein1 spliced
